# Patient and physician perspectives on alopecia areata: A real‐world assessment of severity and burden in Japan

**DOI:** 10.1111/1346-8138.16360

**Published:** 2022-03-28

**Authors:** Emily Edson‐Heredia, Toshihiko Aranishi, Yoshitaka Isaka, Peter Anderson, Simran Marwaha, James Piercy

**Affiliations:** ^1^ Eli Lilly & Company Indianapolis Indiana USA; ^2^ Eli Lilly Japan K.K. Kobe Japan; ^3^ Adelphi Real World Bollington UK

**Keywords:** alopecia areata, dermatology, outcomes research, quality of life, severity of illness

## Abstract

The criteria used by physicians to assess alopecia areata severity and its associated burden from the patients' point of view are not well understood. We aimed to understand physician‐assessed determinants of disease severity, factors associated with severity, patient–physician concordance, and patient‐reported burden by severity. Data were drawn from the Adelphi Alopecia Areata Disease Specific Programme™, a point‐in‐time survey of dermatologists and their alopecia areata patients in real‐world practice in Japan conducted between January and March 2021. Patients were categorized into three groups by current disease severity according to physician subjective assessment: mild, moderate, or severe. Demographics, clinical characteristics, and outcomes were described within and compared between the three patient groups. Our study of 97 dermatologists and 587 patients found overall scalp hair loss was the most important factor considered by physicians in determining disease severity. More severe disease was associated with loss of eyebrow hair, eyelashes, and hair loss from other body areas. Agreement on disease severity between physicians and patients was moderate. From the patient perspective, greater severity of alopecia areata was associated with greater anxiety and depression, with lower work productivity and worse quality of life. Our study provides insights into which factors physicians use to determine alopecia areata severity, how physician and patient severity assessments compare, and the burden of alopecia areata on patients.

## 1 INTRODUCTION

Alopecia areata (AA) is an autoimmune dermatological condition with an often relapsing and remitting course that can be persistent, particularly in cases where there is extensive hair loss.^1^ Although usually manifesting as patchy hair loss on the scalp, AA can also present as total hair loss either on the scalp (alopecia totalis) or across the entire body (alopecia universalis).^2^ AA is one of the most commonly occurring forms of alopecia,^1^, ^3^ estimated to affect 2% of the global population on average,^1^ with onset frequently occurring before 30 years of age.^1^ In some cases, hair can grow back spontaneously in the early stages of disease or when the disease is well controlled, but regrowth is unpredictable and spontaneous. Relapses are also very common, with the proportion of patients likely to see hair regrowth being higher only in those with milder disease.^4^

Alopecia areata is a multifactorial disease, thought to be caused by a combination of genetic and environmental factors. Some researchers have speculated there is a possible association between AA development and certain bacterial and viral infections, psychological stress, and even specific diets;^5^, ^6^, ^7^, ^8^, ^9^ however, many of these studies had small sample sizes and produced mixed findings; therefore, further exploration of the etiology of AA is required. There have also been mixed results reported on the prevalence of AA in men and women, with some studies suggesting AA affects both to a similar extent^10^ while others reported that women were more often affected than men.^11^

The rapid rate of hair loss associated with AA means that patients are at risk of experiencing psychological and psychosocial symptoms, with many AA patients developing low self‐esteem.^5^ Previous research has shown that AA is also associated with anxiety and depression.^12^, ^13^ While AA is likely to have a negative effect on many patients' psychological well‐being, there is some evidence that it may have a greater psychological impact on women,^14^ leading many to have a more negative self‐image,^15^ possibly related to the idea of baldness in women being less acceptable in society than it is in men.^16^ As a result, it is important for health‐care professionals to be aware of the risk of anxiety and depression in AA^12^ and that its effects on quality of life (QoL) may vary across patients.

Extensive AA poses a therapeutic challenge due to lack of efficacy and/or side‐effects of available treatment options, with a lack of approved systemic therapeutics for the disease^17^ and no treatments that induce and sustain remission of AA long term.^18^ For adult patients with limited involvement, intralesional corticosteroids (including triamcinolone acetonide) are considered first‐line therapy, and moderately potent topical steroids are also widely used.^19^ In Japan, approved treatments include topical carpronium chloride hydrate, oral cepharanthine, and the oral combination monoammonium glycyrrhizinate, glycine, and DL‐methionine.^20^, ^21^

The heterogenous nature of AA – with location, pattern and overall percentage hair loss and treatment outcomes often varying across patients – means that AA severity is not easily established. We aimed, therefore, to investigate which features Japanese physicians consider important when determining whether a case of AA is mild, moderate, or severe, as well as to understand the patient‐reported burden of AA in each of these severity groups in real‐world clinical practice in Japan.

## 2 METHODS

### 2.1 Study design

Data were drawn from the Adelphi AA Disease Specific Programme (DSP™), a point‐in‐time survey of dermatologists and their consulting patients conducted in Japan between January and March 2021. The survey captured data on patient demographics as well as current and historical disease severity, and burden and management in a real‐world clinical setting. We used a combination of physician‐ and patient‐reported questionnaires to understand how the disease presented at different degrees of severity and how this impacted QoL from the patient's perspective.

### 2.2 Participants

The dermatologists recruited to participate in this survey reflected a geographically representative sample of the practicing population in Japan. To participate, physicians had to have Japanese Dermatological Association *senmon‐i* certification (demonstrating that the physician is a specialist) and be personally responsible for treatment decisions and the management of patients with AA and consult with a minimum of three adult AA patients monthly, with at least one being currently moderate and one currently severe. Physicians were recruited on a voluntary basis and received an honoraria in line with their time commitment. Once recruited, physicians completed surveys on AA severity and management including percentage of scalp hair loss and other key symptoms they use to diagnose mild, moderate, and severe cases of AA. Each physician then recruited the next three to 10 consecutive consulting adult patients (≥15 years old) with AA, including at least one patient with currently mild and one patient with currently severe AA, with the remaining one to eight being either currently moderate or currently severe. For each patient recruited, the physician completed a patient record form (PRF) based on the details from the patient's medical record as well as information gathered during the consultation.

All patients recruited had a physician‐confirmed diagnosis of AA and were not currently participating in a clinical trial for AA. There were no inclusion restrictions with regards to either the degree of scalp hair loss experienced or treatment received by the patient.

We captured physician‐recorded data on patient demographics and clinical characteristics including AA severity, location, time of onset, and symptoms. AA severity was rated by the physician as either mild, moderate, or severe according to their own definition of these terms based on their clinical experience together with observations, assessments, and medical history of the patient, thus reflecting how physicians classify AA severity in a real‐world clinical setting. We also asked physicians to record the symptom/area affected for each patient and to define the severity of that symptom. Recruited patients were invited to complete a voluntary patient self‐completion form (PSC) at the time of their consultation, independently of the dermatologist. Patients reported their current AA severity based on their own subjective perception of their disease; patient answers could then be matched with corresponding physician‐rated severity to evaluate whether patients and their dermatologists were in alignment on their perception of disease severity. In addition, patients were asked about the impact that AA had on their day‐to‐day activities, work productivity, and emotional and psychological state.

To assess the impact of AA on QoL, the PSC included disease‐specific and general patient‐reported outcome (PRO) tools. The disease‐specific PRO tool used was Skindex‐16 AA, which measured the psychosocial and physical effects of AA. Skindex‐16 is formed of three scales covering patient symptoms (four items), emotions (seven items), and functioning (five items),^22^ and patients can select one of seven answers that lie on a Likert‐type scale ranging from “never bothered” to “always bothered”, with scores varying from 0 (no effect) to 100 (effect experienced all the time). The Hospital Anxiety and Depression Scale (HADS) measures symptoms of generalized anxiety (seven items) and depression (seven items),^23^ with each item being scored 0 to 3, with higher scores indicating greater impact. The EuroQol 5‐dimension 5‐level questionnaire (EQ‐5D‐5L) measures general health status and comprises five dimensions: mobility, self‐care, usual activities, pain/discomfort, and anxiety/depression.^24^ The Work Productivity and Activity Index (WPAI) measures the effect of AA on work productivity during the past 7 days. It comprises questions relating to employment status, hours of work missed, hours actually worked, and productivity while working. WPAI scores range from 0 to 100, with higher scores signifying greater impact of AA on work productivity.^25^

Missing data were not imputed; therefore, the base of patients varies and is thus reported separately for each analysis.

### 2.3 Data analysis

Demographics, clinical/disease characteristics, and PRO were described within and compared between three groups of patients, defined by the overall physician‐rated current AA severity (mild, moderate, severe) of each patient. Statistical tests were conducted to determine whether differences were observed in clinical and demographic characteristics and PRO across current severity groups, where *p* < 0.05 was considered significant. *p*‐values were not multiplicity controlled. Statistical tests used were analysis of variance for numeric variables, and χ^2^‐tests for categorical variables, and Kruskal–Wallis for ordinal variables. Kappa analysis was used to measure patient–physician alignment; kappa scores range from −1 to +1, with below 0.00 indicating poor alignment, 0.00–0.20 slight, 0.21–0.40 fair, 0.41–0.60 moderate, 0.61–0.80 substantial, and 0.81–1.00 almost perfect alignment. Statistical analyses were conducted using STATA version 16.1.^26^

### 2.4 Ethics

The AA DSP received approval from the Western International Review Board in May 2019 (protocol: AG8446). Each survey was performed in full accordance with relevant legislation at the time of data collection, including the US Health Insurance Portability and Accountability Act 1996^27^ and Health Information Technology for Economic and Clinical Health Act legislation.^28^ Data collection was undertaken in line with European Pharmaceutical Marketing Research Association (European Pharmaceutical Market Research Association [EphMRA], September 2019) guidelines^29^ and as such it does not actually require ethics committee approval.

All responses captured on the data collection forms were anonymized to preserve physician and patient confidentiality, and as such no personal identifiable information was requested or collected. All participating physicians and patients were assigned a study number to aid anonymous data collection, and to allow linkage of data during data collection and analysis. Using a check box, patients provided informed consent for use of their anonymized and aggregated data for research and publication in scientific journals.

## 3 RESULTS

### 3.1 Sample size

A total of 97 dermatologists provided PRF data for 587 AA patients, of whom 286 completed a PSC. Physicians rated the current severity of AA as mild for 14% of patients (*n* = 84), moderate for 58% (*n* = 341) of patients, and severe for 28% (*n* = 162) of patients in the sample.

### 3.2 Patient demographics/clinical characteristics

Table 1 shows demographics and clinical characteristics by current AA severity. Patients had a mean age of 43.7 (standard deviation [SD] = 15.4) years with 38% (*n* = 223) being male. We found no significant difference in the mean age of patients across disease severity groups; however, the length of time since diagnosis did differ between groups, with patients with severe AA diagnosed for longer than moderate or mild patients (mean [SD] years: 3.43 [5.86] vs 2.20 [3.10] vs 1.37 [3.07], respectively, *p* = 0.0033). Overall, and within the severity groups, under 4% of patients were reported to have no scalp hair loss; this fell to 1% in patients with moderate or severe AA. Current disease progression differed significantly between severity groups, with physicians reporting a higher percentage of mild patients' AA improving compared with moderate and severe patients. In addition, a higher proportion of patients with severe AA had uncontrolled disease (defined as changeable or worsening either slowly or rapidly; *p* < 0.0001; Table 1).

**TABLE 1:** Patient demographics and clinical characteristics by physician‐rated current severity

	All patients (*n* = 587)	Mild (*n* = 84)	Moderate (*n* = 341)	Severe (*n* = 162)	*p*
Patient demographics					
Age, mean (SD) age	43.7 (15.4)	43.1 (16.3)	44.9 (15.2)	41.6 (15.1)	0.0739^a^
Male sex, *n* (%)	223 (38)	31 (37)	136 (40)	56 (35)	0.5051^b^
BMI, mean (SD)	21.8 (3.1)	20.9 (2.4)	22.0 (3.0)	21.9 (3.4)	**0.0118** ^a^
Clinical characteristics					
Years since diagnosis					
Base, *n*	407	60	241	106	
Mean (SD)	2.40 (4.05)	1.37 (3.07)	2.20 (3.10)	3.43 (5.86)	**0.0033** ^a^
How would you describe this patient's disease progression currently?					
Base, *n*	587	84	341	162	
Improving, *n* (%)	139 (24)	37 (44)	77 (23)	25 (15)	**<0.0001** ^c^
Stable, *n* (%)	268 (46)	35 (42)	164 (48)	69 (43)	
Changeable, *n* (%)	132 (22)	7 (8)	82 (24)	43 (27)	
Worsening slowly, *n* (%)	37 (6)	5 (6)	13 (4)	19 (12)	
Worsening rapidly, *n* (%)	11 (2)	–	5 (1)	6 (4)	
Uncontrolled (changeable and worsening combined), *n* (%)	180 (30)	12 (14)	100 (29)	68 (42)	**<0.0001** ^b^

*Note:* Bold text indicates significant difference across three severity groups (mild, moderate, and severe).

Abbreviations: BMI, body mass index; –, no data available; SD, standard deviations.

^a^ Analysis of variance *F*‐test.

^b^ χ^2^‐test.

^c^ Kruskal–Wallis test.

**FIGURE 1:**
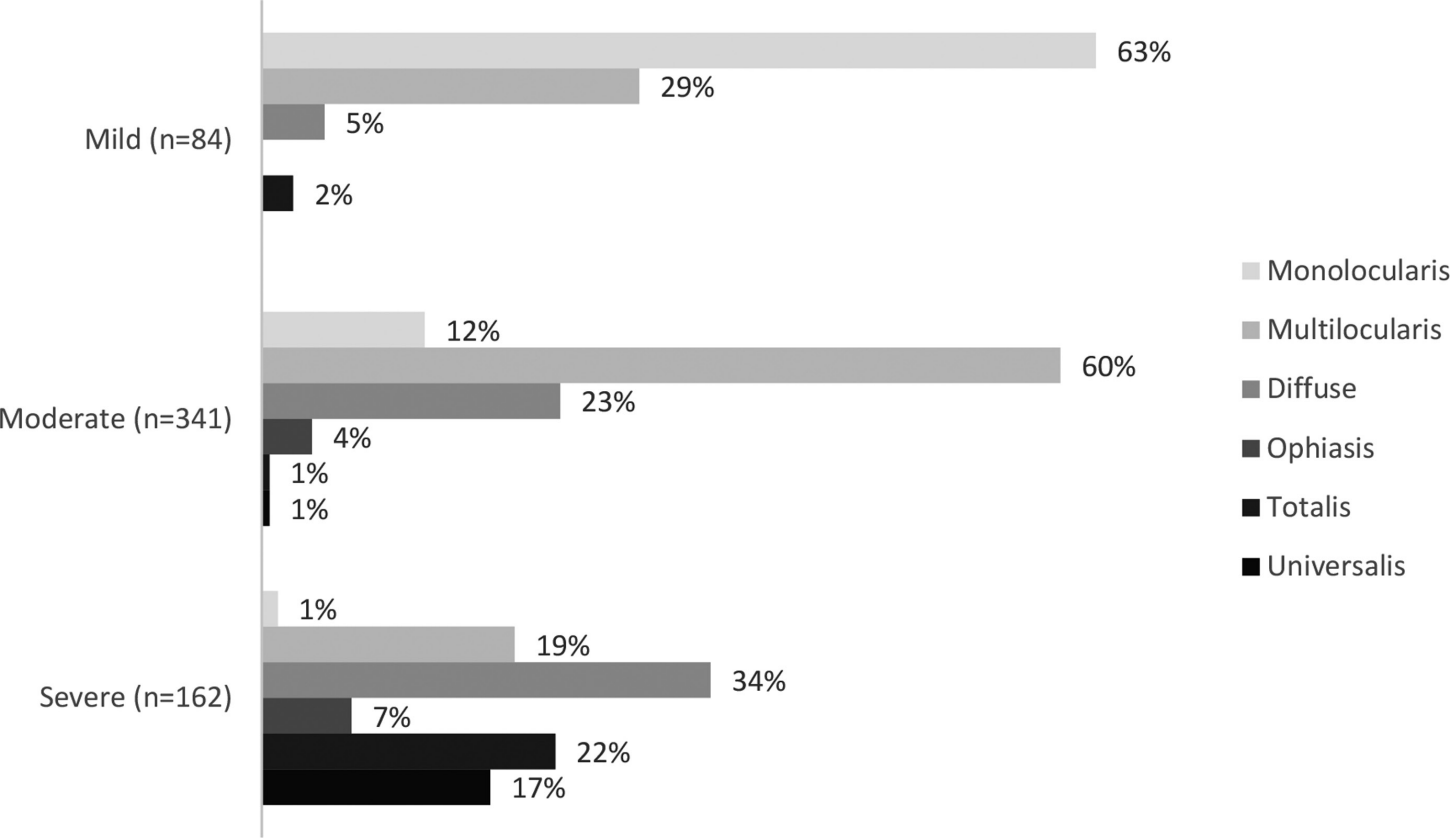
Physician‐reported alopecia areata (AA) type by current severity of patient (physician‐rated)

Figure 1 shows the specific type of AA by current severity. We found that current mild disease was associated with AA monolocularis and AA multilocularis in 63% and 29% of patients, respectively. Current moderate disease was most commonly associated with AA multilocularis (in 60% of patients), followed by AA diffuse and AA monolocularis in 23% and 12% of moderate patients, respectively. Only 1% of moderate patients were labeled as having either AA totalis or AA universalis. Of all AA types, current severe disease was most commonly associated with diffuse (34%), totalis (22%), multilocularis (22%), and universalis (17%), with the majority of patients labeled as AA totalis or universalis being those with severe disease.

In the physician survey, 85% of dermatologists indicated that the most important factor in determining AA severity was the amount of scalp hair loss observed, and 10% reported that patient distress over hair loss was most important.

When surveyed about the percentage of scalp hair loss that would lead them to a diagnosis of mild, moderate, or severe AA, physicians reported a mean percentage (SD) scalp hair loss of 6.9% (4.5) for mild AA, 24.2% (10.7) for moderate AA, and 66.7% (10.99) for severe AA. The actual mean scalp hair loss recorded for this patient cohort was 8.2% in patients with mild AA, 26.2% in moderate, and 72% in patients with severe AA, and therefore was in close agreement with physicians' reported perceptions of disease severity from the survey.

**FIGURE 2:**
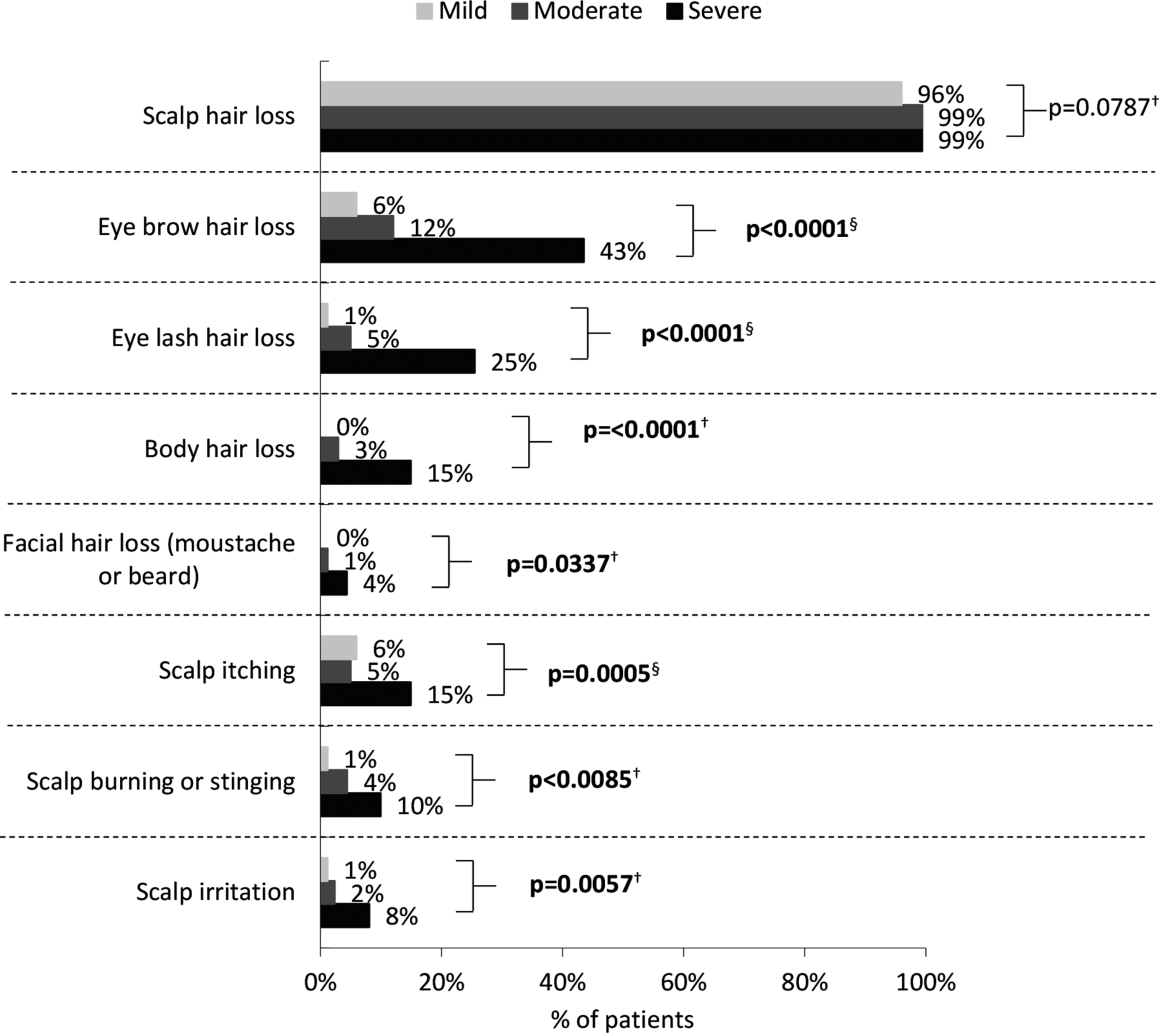
Areas of hair loss by overall current severity of patient (physician‐rated). Significant *p*‐values (<0.05) are shown in bold text. ^†^Fisher's exact test, ^§^χ^2^‐test

Almost all patients in all three severity groups had some degree of scalp hair loss; however, across the severity groups we found a significant difference in the percentage of patients with hair loss in other areas, with patients with severe AA more commonly experiencing eyebrow loss (mild 6% vs moderate 12% vs severe 43%), eyelash loss (mild 1% vs moderate 5% vs severe 25%), and body hair loss (mild 0% vs moderate 3% vs severe 15%) (all *p* < 0.0001; Figure 2).

**FIGURE 3:**
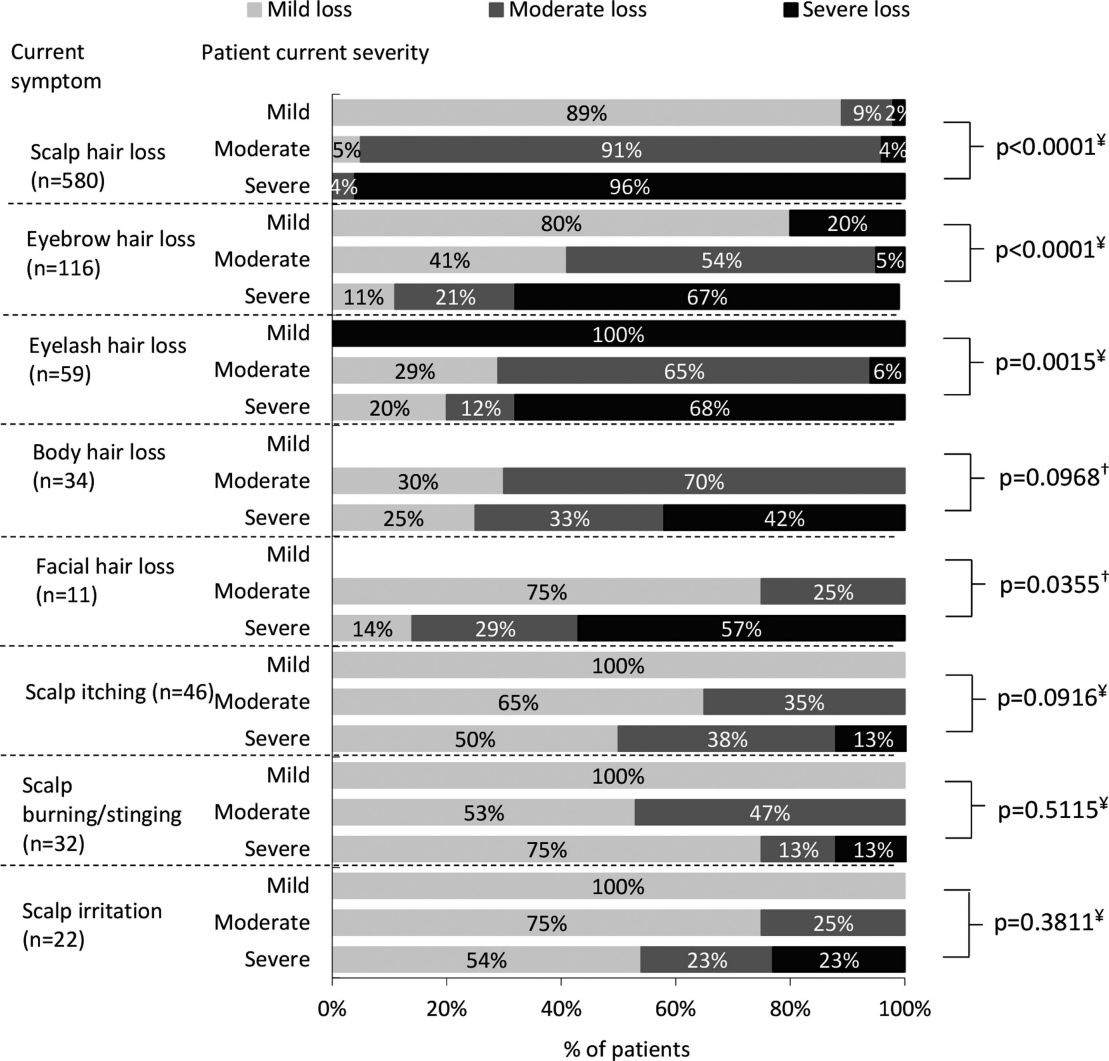
Severity of hair loss in areas affected. ^¥^Kruskal–Wallis test, ^†^Mann–Whitney *U*‐test

When asked to rate the severity of hair loss in each location, physicians reported that for the majority of body areas patient severity was generally aligned with the degree of hair loss in each area affected; that is, hair loss on the scalp, eyebrows, eyelashes, and body was mild in mild patients, while a greater degree of hair loss was observed in these locations in more severe patients. Regarding eyelash loss, whilst only one mild patient was reported to be experiencing this, the hair loss for this patient was classed as severe (Figure 3).

### 3.3 Physician and patient alignment on current disease severity

Table 2 shows the overall alignment of physician‐ and patient‐reported current disease severity. The shaded boxes on the diagonal indicate where the physician and patient were in alignment on assessment of AA severity. In total, we found there was alignment between patient and physician in 76% of cases. In 9% of cases, the patient reported a higher degree of severity than their physician, and in 15% the physician reported higher severity than the patient. Overall, the level of agreement was 0.60 (kappa coefficient) and thus considered to demonstrate a moderate level of agreement between physician and patient (*p* < 0.001).

**TABLE 2:** Alignment of physician‐ and patient‐reported disease severity

*n* = 279		Patient‐reported severity			Kappa coefficient	*p*
Physician‐reported severity	Mild	**27 (10%)**	2 (1%)	2 (1%)	**0.60**	**<0.001**
	Moderate	27 (10%)	**118 (42%)**	20 (7%)		
	Severe	4 (1%)	12 (4%)	**67 (24%)**		

*Note:* Kappa analysis was a measure of agreement between physician and patient using a 6‐point scale, with ratings of 0.0 = poor, 0.0–0.20 = slight, 0.21–0.40 = faint, 0.41–0.60 = moderate, 0.61–0.80 = substantial, and 0.81–1.00 = almost perfect. Bold values indicates significant difference of *p* values.

### 3.4 Patient‐reported outcomes

Table 3 shows that of the 286 patients who completed a PSC, those with severe AA reported higher scores in all three domains of the Skindex‐16 questionnaire (emotions, symptoms, and functioning), indicative of greater number of symptoms, lower functioning, and a higher emotional burden (all *p* < 0.005). Patients with severe AA also reported lower EQ‐5D‐5L scores than those with moderate and mild AA, both overall and within the anxiety/depression dimension; severe patients reported an overall EQ‐5D‐5L mean score of 0.79 compared with 0.90 in mild and 0.87 in moderate patients, indicative of lower overall QoL in patients with severe AA. Similarly, results from the EQ‐5D‐5L anxiety/depression dimension showed a higher percentage of patients with severe AA (73%) reported feeling anxious and/or depressed on the day of their consultation compared with 29% and 42% of patients with mild and moderate AA, respectively (*p* < 0.0001). Patients with severe AA also scored higher on the HADS questionnaire suggesting they had higher levels of generalized anxiety and depression compared with the other severity groups. We also observed a difference in work productivity between the severity groups, with patients with moderate and severe AA reporting significantly greater impairment both while working and in relation to activities outside of work (all *p* < 0.005).

**TABLE 3:** Patient‐reported outcomes by physician‐rated overall severity of AA

	All patients	Mild	Moderate	Severe	*p*
Skindex‐16 emotions					
Base, *n*	286	31	170	85	
Mean	59.9	42.0	56.1	74.0	**<0.0001** ^a^
SD	30.7	33.1	30.2	25.1	
Skindex‐16 symptoms					
Base, *n*	284	31	168	85	
Mean	20.1	12.5	18.0	27.1	**0.0018** ^a^
SD	23.2	16.8	22.3	25.4	
Skindex‐16 functioning					
Base, *n*	285	31	169	85	
Mean	45.5	26.3	40.2	63.2	**<0.0001** ^a^
SD	32.3	31.3	31.0	27.4	
EQ‐5D‐5L					
Base, *n*	286	31	170	85	
Mean	0.85	0.90	0.87	0.79	**<0.0001** ^a^
SD	0.12	0.05	0.10	0.13	
EQ‐5D‐5L: Anxiety/depression dimension: Please indicate which statements best describe your health today					
Base, *n*	286	31	170	85	
I am anxious or depressed, *n* (%)	142 (50)	9 (29)	71 (42)	62 (73)	**<0.0001** ^b^
Hospital Anxiety and Depression Scale (HADS)					
Depression score, mean (SD)	5.36 (4.42)	3.74 (4.01)	4.99 (4.45)	6.63 (4.22)	**0.0039** ^a^
Anxiety score, mean (SD)	6.21 (4.61)	4.56 (3.87)	5.78 (4.7)	7.62 (4.35)	**0.0024** ^a^
WPAI: Percent work time missed due to problem					
Base, *n*	173	19	105	49	0.5538^a^
Mean	1.2	2.0	1.0	1.4	
SD	3.9	4.2	4.2	3.2	
WPAI: Percent impairment while working due to problem					
Base, *n*	173	19	103	51	**0.0002** ^a^
Mean	23.5	13.2	19.6	35.3	
SD	25.1	14.2	24.2	26.41	
WPAI: Percent overall work impairment due to problem					
Base, *n*	171	19	103	49	**0.0003** ^a^
Mean	24.1	14.7	20.2	35.8	
SD	25.4	15.5	24.7	26.5	
WPAI: Percent activity impairment due to problem					
Base, *n*	285	31	169	85	**<0.0001** ^a^
Mean	33.4	13.2	28.3	51.9	
SD	28.5	13.8	26.2	27.2	

*Note:* Bold text indicates significant difference across the severity groups (mild, moderate, and severe).

Abbreviations: AA, alopecia areata; EQ‐5D‐5L, EuroQol 5‐Dimension 5‐level questionnaire; SD, standard deviations; WPAI, Work Productivity and Activity Impairment.

^a^ Analysis of variance *F*‐test.

^b^ χ^2^‐test.

## 4 DISCUSSION

Owing to limited Japan‐specific information on the prevalence and burden of AA by severity, this paper fills an important gap in knowledge. We found that patients with more severe AA experienced worse patient‐reported burden, with more severe patients reporting worse AA symptoms, more negative emotions, and greater impairment of functioning when undertaking everyday activities. A separate study of AA patients in Japan reported 40% of the study cohort at risk for depressive disorders;^30^ our results build on their findings, showing that overall quality of life was lower in patients with severe AA, and the prevalence of anxiety and depression was higher compared with patients with current mild or moderate disease. Patients with severe AA also reported greater impairment both at work and in relation to activities outside of work. It was also observed that activity impairment was correlated with increasing severity. Our findings were similar to those of other studies; such as Abedini *et al*. who also suggested that AA had a considerable impact on QoL and that this was more pronounced in patients with severe disease.^31^ Specifically, while we observed anxiety and depression in many AA patients, prevalence was greater in more severe cases. It should also be noted that our findings in Japan were consistent with those in a study in the USA using an identical methodology (Burge R *et al*. 2021, unpublished data).

When asked which factors drive the definition of mild, moderate, or severe AA, physicians reported the degree of scalp hair loss as being the main indicator of severity followed by patient distress over hair loss. Previous studies have also highlighted area of scalp hair loss as a key indicator of AA severity^32^ as well as an important driver of therapy selection.^33^ We also found a greater degree of body, eyelash, and eyebrow hair loss in severe AA patients compared with moderate and mild patients.

We observed a moderate level of physician–patient alignment on disease severity; this was likely due to AA being a very visible disease, often affecting the patient's head and face, and thus allowing physicians to make a quick and accurate assessment of the locations affected and the extent of hair loss during the consultation. A previous study by *Reid et al*. reported lower levels of patient–physician alignment than found in our study, with some patients reporting the extent of their hair loss as more severe than that which was reported by the physician.^34^ Reid *et al*. also found that patient‐reported QoL corresponded more closely with the patient's own perception of hair loss rather than that of their physician. They suggested that the difference in physician and patient perception might be due to the negative impact the hair loss had on patients' self‐image causing them to have a distorted view of how bad the hair loss was.^34^ This study differed from ours in that it included only female patients. While there is evidence to suggest that women in particular experience negative psychosocial effects from hair loss,^16^ it is unlikely that the mismatch between extent of hair loss and patient burden is exclusive to women; for example, a study on both male and female patients in Japan also reported that objective severity did not necessarily correlate with the effect of the disease on patient QoL.^30^ Thus, it may not be sufficient to consider the psychosocial impact on the patient solely in terms of the degree of hair loss. When treating these patients, it is therefore important for physicians to be aware of the psychosocial impact associated with AA as low self‐esteem and depression may negatively influence the patients' own perception of their disease. In cases of disconnect between patients and physicians, improved communication will be key as there is evidence that good patient experience can lead to better engagement with treatment^35^ and promote adherence to prescribed therapies.^36^

We reported that fewer patients with severe AA were improving compared with those who have mild and moderate AA and a higher percentage of patients with severe AA were uncontrolled, with changeable or worsening disease. These findings were similar to a previous study that reported unstable disease course as being associated with more severe disease.^31^

## CONFLICT OF INTEREST

Emily Edson‐Heredia, Toshihiko Aranishi, and Yoshitaka Isaka are employees and stockholders of Eli Lilly and Company. Peter Anderson, James Piercy, and Simran Marwaha are employees of Adelphi Real World, a research organization who conducted the study under contract by Eli Lilly and Company.
